# Patients lacking classical poor prognostic markers might also benefit from a step-down glucocorticoid bridging scheme in early rheumatoid arthritis: week 16 results from the randomized multicenter CareRA trial

**DOI:** 10.1186/s13075-015-0611-8

**Published:** 2015-04-09

**Authors:** Patrick Verschueren, Diederik De Cock, Luk Corluy, Rik Joos, Christine Langenaken, Veerle Taelman, Frank Raeman, Isabelle Ravelingien, Klaas Vandevyvere, Jan Lenaerts, Elke Geens, Piet Geusens, Johan Vanhoof, Anne Durnez, Jan Remans, Bert Vander Cruyssen, Els Van Essche, An Sileghem, Griet De Brabanter, Johan Joly, Kristien Van der Elst, Sabrina Meyfroidt, Rene Westhovens, on behalf of the CareRA study group

**Affiliations:** Skeletal Biology and Engineering Research Center, KU Leuven Department of Development and Regeneration, Herestraat 49, 3000 Leuven, Belgium; Department of Rheumatology, University Hospitals Leuven, Herestraat 49, 3000 Leuven, Belgium; Reuma-Instituut Hasselt, Anne Frankplein 17, 3500 Hasselt, Belgium; Jessa Ziekenhuis Hasselt, Stadsomvaart 11, 3500 Hasselt, Belgium; ZNA Jan Palfijn Antwerpen, Lange Bremstraat 70, 2170 Merksem, Belgium; Heilig Hart Ziekenhuis Leuven, Naamsestraat 105, 3000 Leuven, Belgium; Department of Rheumatology, Onze-Lieve-Vrouw Ziekenhuis Aalst, Bloklaan 5, 1730 Asse Aalst, Belgium; AZ Groeninge Hospital Kortrijk, Pres. Kennedylaan 4, 8500 Kortrijk, Belgium; ReumaClinic Genk & UHasselt, Jaarbeurslaan 21, 3600 Genk, Belgium; Maastricht UMC, P. Debyelaan 25, 6229 HX Maastricht, the Netherlands; Reuma-Instituut Genk, Weg naar As 123, 3600 Genk, Belgium; Imeldaziekenhuis Bonheiden, Imeldalaan 9, 2820 Bonheiden, Belgium; ReumaClinic Hasselt, Jaarbeurslaan 21, 3600 Genk, Belgium; AZ Sint Lucas Brugge, Sint-Lucaslaan 29, 8310 Brugge, Belgium; Skeletal Biology and Engineering Research Center, KU Leuven Department of Public Health and Primary Care, Herestraat 49, 3000 Leuven, Belgium

## Abstract

**Introduction:**

Considering a lack of efficacy data in patients with early rheumatoid arthritis (eRA) presenting without classical markers of poor prognosis, we compared methotrexate (MTX) with or without step-down glucocorticoids in the CareRA trial.

**Methods:**

Disease-modifying antirheumatic drug–naïve patients with eRA were stratified into a low-risk group based on prognostic markers that included non-erosiveness, anti–citrullinated protein antibodies and rheumatoid factor negativity and low disease activity (Disease Activity Score in 28 joints based on C-reactive protein (DAS28(CRP)) ≤3.2). Patients were randomized to 15 mg of MTX weekly (MTX with tight step-up (MTX-TSU)) or 15 mg of MTX weekly with prednisone bridging, starting at 30 mg and tapered to 5 mg daily from week 6 (COmbinatie therapie bij Reumatoïde Artritis (COBRA Slim)). A TSU approach was applied. Outcomes assessed were DAS28(CRP)-determined remission, cumulative disease activity, Health Assessment Questionnaire (HAQ) scores and adverse events (AEs) after 16 treatment weeks.

**Results:**

We analyzed 43 COBRA Slim and 47 MTX-TSU patients and found that 65.1% in the COBRA Slim group and 46.8% in the MTX-TSU group reached remission (*P* = 0.081). Mean ± standard deviation area under the curve values of DAS28(CRP) were 13.84 ± 4.58 and 11.18 ± 4.25 for the MTX-TSU and COBRA Slim patients, respectively (*P* = 0.006). More COBRA Slim patients had an HAQ score of 0 (51.2% versus 23.4%, *P* = 0.006) at week 16. Therapy-related AEs between groups did not differ.

**Conclusion:**

In patients with low-risk eRA, MTX with step-down glucocorticoid bridging seems more efficacious than MTX step-up monotherapy, with a comparable number of AEs observed over the first 16 treatment weeks.

**Trial registration:**

EU Clinical Trials Register Identifier: EudraCT number 2008-007225-39. Registered 5 November 2008.

**Electronic supplementary material:**

The online version of this article (doi:10.1186/s13075-015-0611-8) contains supplementary material, which is available to authorized users.

## Introduction

Current guidelines recommend treating patients with early rheumatoid arthritis (eRA) immediately, intensively and to target [1-3]. Early intensive treatment strategies combining classical disease-modifying antirheumatic drugs (DMARDs) with rapid remission-inducing agents such as glucocorticoids (GCs) or biologicals are the most effective approach for eRA [4-6]. In daily practice, however, the initial treatment choice is based on the physician’s preference, patient and disease characteristics and cost issues [7]. Traditionally, the absence of bone erosions, rheumatoid factor (RF) or anti–citrullinated protein antibodies (ACPA) and low disease activity are considered markers of a good prognosis, but the bad performance of these markers and derived matrices might lead to undertreatment of so-called low-risk patients [8].

New, very sensitive classification criteria for rheumatoid arthritis (RA) were developed in light of the early treatment paradigm [9], but patients with eRA still form a heterogeneous group [10]. Current treatment recommendations are mostly based on evidence from randomized controlled trials (RCTs) in preselected populations with a poor prognosis based on classical markers and high disease activity. In few studies have researchers examined how to treat patients in a way not reflective of this classic RCT image of eRA.

Early intensive treatment appears successful also in patients with undifferentiated arthritis, including patients with so-called pre-RA, but confirmation is needed in studies with a longer follow-up [11]. Some authors, however, suggest that too stringent treatment targets might not outweigh the potential side effects in patients with eRA lacking markers of poor prognosis [12].

In this study, we evaluated the efficacy and safety of step-up methotrexate (MTX) with or without a step-down GC bridging scheme after 16 weeks of treatment in patients with eRA presenting without classical markers of poor prognosis.

## Methods

This study is part of the Care for Early RA (CareRA) trial, a Flemish, prospective, 2-year, investigator-initiated, multicenter RCT rooted in daily practice (EudraCT number 2008-007225-39). The trial is conducted in two academic centers, seven general hospitals and four private practices.

The ethics committee (EC) of the University Hospitals Leuven approved this study after consultation with the local ECs. All patients gave us their written informed consent to participate. The full names of all approving ECs are provided in the Acknowledgements section.

### Patients

DMARD-naïve patients with eRA, as defined by the American College of Rheumatology 1987 criteria [13], aged ≥18 years and with a disease duration ≤1 year, were recruited between January 2009 and May 2013. Patients having contraindications for MTX and/or GCs were excluded. Additional file 1 describes the exclusion criteria in more detail.

Eligible patients were stratified into a low- or high-risk group. This allocation was based on classic RA prognostic factors: presence of erosions, presence of RF or ACPA, and baseline Disease Activity Score in 28 joints based on C-reactive protein (DAS28(CRP)).

Patients were considered low risk if they satisfied one of the following combinations of severity markers:

- No erosions + ACPA- and RF-negative
- Erosions + ACPA- and RF-negative + DAS28(CRP) ≤3.2
- No erosions + ACPA- and/or RF-positive + DAS28(CRP) ≤3.2

**Figure 1:**
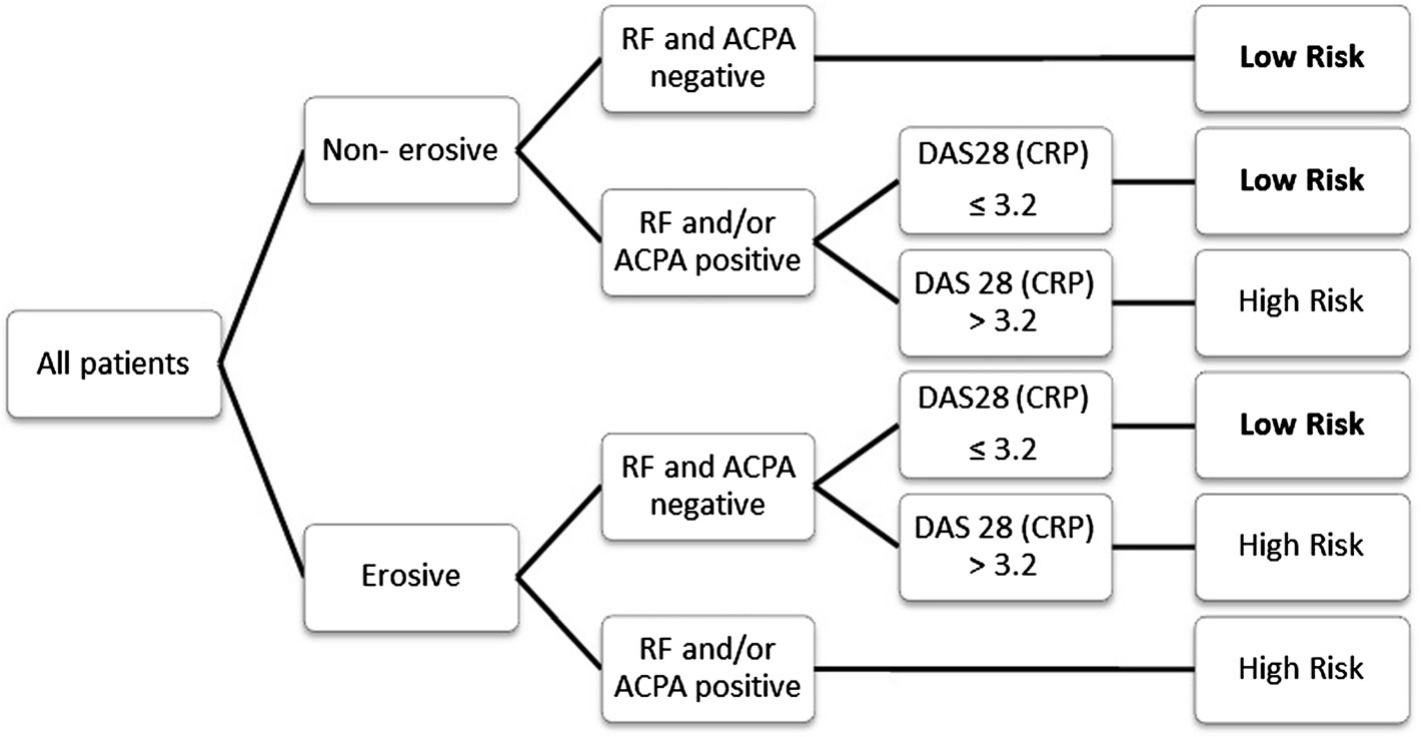
Classification of patients into high- or low-risk group according to classic prognostic factors. ACPA, Anti–citrullinated protein antibodies; DAS28(CRP), Disease Activity Score in 28 joints based on C-reactive protein; RF, Rheumatoid factor.

See Figure 1 for more detail about the risk stratification.

Patients were assessed at screening, baseline, week (W)4, W8 and W16. If a treatment adjustment was required at W8, an optional visit was held at W12. The analysis of the first 16 weeks in the high-risk arm of the CareRA trial was previously reported [14].

### Design

Low-risk patients were randomized to one of two treatment arms:

1. MTX tight step-up (MTX-TSU): 15 mg of MTX weekly, no oral steroids allowed
2. COmbinatie therapie bij Reumatoïde Artritis (COBRA Slim): 15 mg of MTX weekly with a step-down scheme of daily oral GCs (30–20–12 mg, 5-10-7.5 and 5 mg of prednisone). From W28, GCs were tapered on a weekly basis by leaving out one daily dose each week over a period of 6 weeks until complete discontinuation.

A treat-to-target approach was used in a tight control setting [15], aiming for a DAS28(CRP) ≤3.2. If patients failed to reach this goal, treatment adjustments were made in both groups from W8: first a MTX dose increase to 20 mg weekly and then the addition of 10 mg of leflunomide daily. Not reaching the target after these treatment adjustments was considered an efficacy failure. Intramuscular and intraarticular GC injections were allowed maximally every 8 weeks, except within 4 weeks preceding W16.

### Outcome

The primary outcome was the proportion of patients in remission at W16, defined as a DAS28(CRP) <2.6. Secondary outcomes were the proportion of good responders according to European League Against Rheumatism (EULAR) criteria [9], patients having a clinically meaningful Health Assessment Questionnaire (HAQ) response, patients having a HAQ score of 0 at W16 and cumulative disease activity.

### Safety and toxicity

Patients were asked about experienced adverse events (AEs) at each visit. Each reported AE was subtyped (toxicity, discomfort, infection, surgery or other) and evaluated for relationship to the therapy, seriousness and severity by the treating rheumatologist. In cases of toxicity, medication was adjusted according to a predefined scheme. Persistent toxicity was considered a safety failure.

### Statistical analysis

No power calculation was done, in view of the low-risk subanalysis of the CareRA trial.

We performed an intention-to-treat (ITT) analysis by χ^2^ test, Mann–Whitney *U* test, area under the curve (AUC) and generalized estimating equation (GEE) analysis. Screening data were used to impute missing baseline data and vice versa. A maximum likelihood model was applied to impute missing data at W4, W8 and W16. Missing data at the optional visit W12 were imputed by taking the mean of W8 and W16. SPSS version 20.0 software (IBM, Armonk, NY, USA) was used. A *P*-value <0.05 was considered statistically significant.

## Results

A group of 90 of the 380 patients in the CareRA trial were stratified as low-risk patients, comprising 47 MTX-TSU and 43 COBRA Slim patients. Both of these subgroups had similar baseline characteristics, which reflect a mild eRA, with a moderate mean disease activity and low numbers for erosions, RF and ACPA positivity (Table 1). One MTX-TSU and three COBRA Slim patients withdrew their consent before W16.

**Table 1:** **Patients’ characteristics at baseline per treatment group** ^**a**^

	**MTX-TSU**	**COBRA Slim**
	**n = 47**	**n = 43**
Age (yr)	51.02 ± 14.00	51.42 ± 14.42
BMI (kg/m^2^)	26.98 ± 4.22	25.40 ± 4.27
Female sex	80.9%	76.7%
Smoking status (ever)	38.3%	48.2%
Alcohol intake (yes)	61.7%	55.8%
Symptom duration (wk)	33.11 ± 62.21	34.42 ± 68.16
Disease duration (wk)	3.17 ± 6.62	1.86 ± 2.70
Employed before onset (yes)	66.0%	55.8%
Currently employed (yes)	57.4%	51.2%
Comorbidities present (yes)	66.0%	60.5%
Nocturnal pain (yes)	72.3%	48.8%
Morning stiffness (yes)	68.1%	53.5%
RF (yes)	23.4%	25.6%
Anti-CCP (yes)	23.4%	27.9%
Erosions (yes)	0.0%	2.3%
Total TJC	14.06 ± 8.61	13.14 ± 10.70
Total SJC	10.00 ± 6.98	10.93 ± 7.55
TJC28	9.49 ± 7.46	8.51 ± 7.80
SJC28	6.89 ± 6.11	7.79 ± 6.03
PGA (range: 0 to 100)	49.89 ± 22.99	48.60 ± 30.68
Pain (range: 0 to 100)	52.09 ± 23.23	48.23 ± 31.19
Fatigue (range: 0 to 100)	45.91 ± 22.07	39.40 ± 27.66
PhGA (range: 0 to 100)	48.34 ± 23.37	48.63 ± 20.80
ESR (mm/hr)	23.04 ± 16.90	30.00 ± 29.40
CRP (mg/L)	13.53 ± 18.62	20.14 ± 39.25
DAS28(ESR)	4.83 ± 1.68	4.88 ± 1.64
DAS28(CRP)	4.55 ± 1.63	4.50 ± 1.63
HAQ (range: 0 to 3)	0.99 ± 0.67	0.92 ± 0.85

^a^Alcohol intake, Consumption of any form of alcohol; Anti-CCP, Anti–citrullinated protein antibodies; BMI, Body mass index; COBRA Slim, COmbinatie therapie bij Reumatoïde Artritis; CRP, C-reactive protein; DAS28, Disease Activity Score in 28 joints; Disease duration, Time elapsed between diagnosis of RA and start of treatment; ESR, Erythrocyte sedimentation rate; HAQ, Health Assessment Questionnaire; Morning stiffness, Being stiff in the morning for at least 45 minutes; MTX-TSU, Methotrexate with tight step-up, 15 mg of MTX weekly, no oral steroids allowed; PGA, Patient global assessment; PhGA, Physician global assessment; RF, Rheumatoid factor; SJC, Swollen joint count; Symptom duration, Time elapsed between onset of symptoms and start of treatment; TJC, Tender joint count. Values reported are proportions or mean ± standard deviation.

### Efficacy

#### Primary and secondary outcomes

Remission was accomplished in 46.8% of MTX-TSU patients and 65.1% of COBRA Slim patients (*P* = 0.081). A good EULAR-defined response was achieved in 44.7% of MTX-TSU and 58.1% of COBRA Slim patients (*P* = 0.202). A clinically meaningful HAQ response was reached in 53.2% of MTX-TSU patients and 62.8% of COBRA Slim patients (*P* = 0.357). Fewer patients had a HAQ score of 0 in the MTX-TSU group (23.4%) than in the COBRA Slim group (51.2%) (*P* = 0.006). Table 2 describes these outcomes in more detail.

**Table 2:** **Clinical outcomes at week 16 per treatment group** ^**a**^

	**MTX-TSU**	**COBRA Slim**	***P*** **-value**
	**n = 47**	**n = 43**	
DAS28(CRP) change	1.76 ± 1.68	2.12 ± 1.41	0.192
Remission	46.8%	65.1%	0.081
Low disease activity	72.3%	79.1%	0.458
Good EULAR response	44.7%	58.1%	0.202
Moderate EULAR response	72.3%	86.0%	0.111
HAQ change	0.40 ± 0.62	0.58 ± 0.64	0.267
Clinically meaningful HAQ change	53.2%	62.8%	0.357
HAQ score = 0	23.4%	51.2%	0.006

^a^COBRA Slim, COmbinatie therapie bij Reumatoïde Artritis; DAS28(CRP), Disease Activity Score in 28 joints based on C-reactive protein; DAS28(CRP) change, Disease Activity Score in 28 joints at baseline minus Disease Activity Score in 28 joints at week 16; EULAR, European League Against Rheumatism; HAQ, Health Assessment Questionnaire; HAQ change, Baseline Health Assessment Questionnaire score minus week 16 Health Assessment Questionnaire score; MTX-TSU, Methotrexate with tight step-up, 15 mg of MTX weekly, no oral steroids allowed. Clinically meaningful HAQ change is defined as a Health Assessment Questionnaire score change >0.22. Remission is defined as DAS28(CRP) <2.6. Low disease activity is defined as DAS28(CRP) ≤3.2. Good EULAR response is defined as low disease activity with a DAS28(CRP) change >1.2. Moderate EULAR response is defined as DAS28(CRP) change >1.2 or DAS28(CRP) change ≤5.1 and a DAS28(CRP) change between 0.6 and 1.2. Values reported are proportions or mean ± standard deviation. χ^2^ tests and Mann–Whitney *U* tests were applied when appropriate. The significance level was set at 0.05.

#### Longitudinal analyses

**Figure 2:**
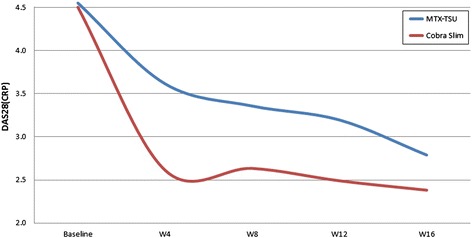
Areas under the curve of Disease Activity Score in 28 joints based on C-reactive protein in each treatment group. COBRA Slim, COmbinatie therapie bij Reumatoïde Artritis; DAS28(CRP), Disease Activity Score in 28 joints based on C-reactive protein; MTX-TSU, 15 mg of MTX weekly with tight step-up; W, Week.

The mean ± SD AUC DAS28(CRP) was 13.84 ± 4.58 and 11.18 ± 4.25 for the MTX-TSU and COBRA Slim patients, respectively (*P* = 0.006) (Figure 2). GEE analysis showed a better treatment effect on longitudinal disease activity of COBRA Slim compared with MTX-TSU (*P* = 0.005).

#### Treatment adaptations

At W8, treatment adjustments were performed in 34.0% of MTX-TSU patients and 23.3% of COBRA Slim patients (*P* = 0.259). At W16, treatment adjustments were performed in 21.3% of MTX-TSU patients and 16.3% of COBRA Slim patients (*P* = 0.545). One COBRA Slim patient was considered to have experienced an efficacy failure at W16.

Intraarticular GC injections were given in 21.3% of MTX-TSU patients and 7.0% of COBRA Slim patients (*P* = 0.054). Only one MTX-TSU patient received two GC injections.

### Safety

Until W16, therapy-related AEs were reported in 44.7% of MTX-TSU patients and in 39.5% of COBRA Slim patients (*P* = 0.622). MTX-TSU was related to 32 AEs and COBRA Slim to 30 AEs, with a similar distribution for discomfort and toxicity (Table 3). In the MTX-TSU group, 11 of 23 AEs related to discomfort were intestinal problems (nausea and diarrhea), and 10 of 23 discomfort problems in the COBRA Slim group were intestinal issues (nausea and constipation). In the COBRA Slim group, there were two cases of increased appetite. Furthermore, 8 of 23 discomfort problems in the MTX-TSU group and 8 of 23 AEs related to discomfort in the COBRA Slim group were general malaise problems (dizziness, agitation, headache and fatigue). There were seven toxicity problems related to therapy in the MTX-TSU group, comprising four cases of abnormal liver values, one of abnormal kidney values, one of oral ulcer and one of pyrosis. In the COBRA Slim group, there were four toxicity problems related to therapy, comprising two cases of alopecia, one of tendinitis and one of stomatitis. The only infection in our study was an upper respiratory tract infection in a MTX-TSU patient. No serious AEs were registered. Additional file 2 gives a detailed overview of the comorbidities.

**Table 3:** **Number of adverse events per treatment group** ^**a**^

		**MTX-TSU**	**COBRA Slim**
		**n = 47**	**n = 43**
AEs related to therapy		32	30
Type of related AEs		23	23
	Discomfort		
	Toxicity	7	4
	Infection	1	0
	Others	1	3
	Surgery	0	0
Severity of related AEs		29	28
	Mild		
	Moderate	3	2
	Severe	0	0
Serious AEs		0	0

^a^AE, Adverse event; COBRA Slim, COmbinatie therapie bij Reumatoïde Artritis; MTX-TSU, Methotrexate with tight step-up, 15 mg of MTX weekly, no oral steroids allowed. Protocol determines the severity rating of the adverse event: mild (does not interfere with daily living), moderate (somewhat interferes with daily living or medications needed to relieve event) or severe (incapacitating).

Weight gain (mean ± SD) was 0.00 ± 2.44 kg in the MTX-TSU group and 0.70 ± 3.16 kg in the COBRA Slim group (*P* = 0.287). Body mass index gain (mean ± SD) was 0.01 ± 0.90 kg/m^2^ in the MTX-TSU group and 0.23 ± 1.12 kg/m^2^ in the COBRA Slim group (*P* = 0.286).

## Discussion

We have demonstrated that, although the primary outcome was not met at W16, low-risk patients with eRA treated with MTX and a step-down GC bridging scheme showed a better cumulative control of disease activity over time and better functionality than patients treated with step-up MTX only, with a similar safety profile maintained during the first 16 treatment weeks.

In both groups, favorable remission and low disease activity scores were achieved after 16 weeks. Efficacy scores did not differ at W16, which is probably due to the limited number of patients included in this substudy, as well as to a trend for more treatment modifications and GC injections in the MTX-TSU group. The lower cumulative disease activity in the COBRA Slim group during the first 16 weeks of treatment might have important consequences for the future disease course [16,17]. Moreover, the speed of disease control and frequency in treatment adaptations could also have differential effects on the evolution of patient-centered outcomes.

In this study, we applied a step-down bridge GC scheme that has two advantages over more traditional, short-term, low-dose GC use [18]. First, high-dose or moderately dosed GCs demonstrate, apart from slow genomic effects, faster nongenomic effects, with a more profound impact on the disease process [19,20]. Second, systematic and prolonged use of GCs is more efficacious than on-demand use in the therapeutic time window before maximum DMARD efficacy [6,21]. Intensive remission induction regimens in so-called low-risk patients with eRA appear to be equally advantageous as in high-risk patients with eRA, but the appropriateness and performance of the currently used prognostic parameters need further evaluation in the long term [8].

A significant finding is the safety profile of both groups. Patients in the MTX-TSU and COBRA Slim groups showed comparable numbers and types of AEs related to therapy in the period during which their treatment schedules differed the most. Not much is known about the safety of short-term GC use. Our study adds to the much-needed evidence about GC use in the management of eRA [22,23] and shows that GC s are relatively safe to use in a remission induction scheme in patients with eRA, as well as in so-called mild RA. This result is in contrast to some rheumatologists’ negative perception of GC use in intensive treatment strategies in eRA [24], whereas patients themselves are rapidly convinced after GC administration [25].

This exploratory study has some limitations. First, the total population in the low-risk arm was relatively small. Power calculation for the CareRA study was done in view of the high-risk subpopulation. Because 25% of patients were stratified as low-risk patients, we could not reach the same power as in the high-risk arm. Therefore, the results of our explorative study in the low-risk arm should be interpreted with caution. Furthermore, the low number of low-risk patients may be responsible for the lack of statistical difference in the primary outcome at W16. Second, we did not measure medication adherence, and there was no blinding procedure, but this is unavoidable in a pragmatic trial reflecting daily clinical practice.

Third, we report the results after 16 weeks of treatment, which is a relatively short time span in which to evaluate the full impact of a treatment strategy. This timing was chosen because there is increasing evidence that long-term RA outcomes are mostly influenced by the initial success of treatment. Of course, the ultimate effect of treat-to-target adaptations according to the protocol cannot be evaluated within this time window.

Our exploratory data are of importance in the ongoing debate about the optimal initial treatment strategy for eRA in daily practice [26]. Patients with RA who are negative for biomarkers such as RF and especially ACPA are traditionally seen as having a better prognosis. Barra *et al*. showed very clearly that this assumption is not always true [27]. The absence of serum markers for RA cannot be claimed to predict in general a milder disease course. This means that patients conventionally perceived as having a lower risk of a severe disease course should be treated according to the same standards as high-risk patients. In this study, we show that, just like high-risk patients, so-called low-risk patients can be more successfully treated with an intensive treatment strategy while having a similar safety outcome as patients treated more conservatively. Until such time that prognostic factors can reliably stratify patients by prognosis to specific treatment approaches, our data suggest every patient with RA could benefit from an upfront intensive treatment approach.

## Conclusions

Patients with eRA perceived to be at low risk of a severe disease course seem to improve more, at least in terms of cumulative disease activity and functionality, if treated intensively with MTX and a step-down bridge moderate-dose GC scheme compared with MTX alone over the course of 16 weeks.

## Additional files

Additional file 1:
**List of exclusion criteria in the CareRA trial.**
Additional file 2:
**List of comorbidities in the study population and per treatment category.**

## Abbreviations

ACPA: Anti–citrullinated protein antibodies
AE: Adverse event
AUC: Area under the curve
BMI: Body mass index
CareRA: Care in Early RA
COBRA Slim: COmbinatie therapie bij Reumatoïde Artritis
DAS28(CRP): Disease Activity Score in 28 joints based on C-reactive protein
DMARD: Disease-modifying antirheumatic drug
EC: Ethics committee
eRA: Early rheumatoid arthritis
ESR: Erythrocyte sedimentation rate
EULAR: European League Against Rheumatism
GC: Glucocorticoid
GEE: Generalized estimating equation
ITT: Intention to treat
MTX: Methotrexate
PGA: Patient global assessment
PhGA: Physician global assessment
RA: Rheumatoid arthritis
RCT: Randomized controlled trial
RF: Rheumatoid factor
SD: Standard deviation
SJC: Swollen joint count
TJC: Tender joint count
TSU: Tight step-up

## References

[1] Smolen JS, Landewé R, Breedveld FC, Buch M, Burmester G, Dougados M (2014). EULAR recommendations for the management of rheumatoid arthritis with synthetic and biological disease-modifying antirheumatic drugs: 2013 update. Ann Rheum Dis..

[2] Singh JA, Furst DE, Bharat A, Curtis JR, Kavanaugh AF, Kremer JM (2012). 2012 update of the 2008 American College of Rheumatology recommendations for the use of disease-modifying antirheumatic drugs and biologic agents in the treatment of rheumatoid arthritis. Arthritis Care Res..

[3] Deighton C, O’Mahony R, Tosh J, Turner C, Rudolf M, Guideline Development Group (2009). Management of rheumatoid arthritis: summary of NICE guidance. BMJ.

[4] Boers M, Verhoeven AC, Markusse HM, van de Laar MA, Westhovens R, van Denderen JC (1998). Randomised comparison of combined step-down prednisolone, methotrexate and sulphasalazine with sulphasalazine alone in early rheumatoid arthritis. Lancet. 1997;350:309–18. A published erratum appears in. Lancet.

[5] Möttönen T, Hannonen P, Leirisalo-Repo M, Nissilä M, Kautiainen H, Korpela M, the FIN-RACo trial group (1999). Comparison of combination therapy with single-drug therapy in early rheumatoid arthritis: a randomised trial. Lancet.

[6] Goekoop-Ruiterman YP, de Vries-Bouwstra JK, Allaart CF, van Zeben D, Kerstens PJ, Hazes JM (2007). Comparison of treatment strategies in early rheumatoid arthritis: a randomized trial. Ann Intern Med..

[7] De Cock D, Vanderschueren G, Meyfroidt S, Joly J, Westhovens R, Verschueren P (2014). Two-year clinical and radiologic follow-up of early RA patients treated with initial step up monotherapy or initial step down therapy with glucocorticoids, followed by a tight control approach: lessons from a cohort study in daily practice. Clin Rheumatol..

[8] De Cock D, Vanderschueren G, Meyfroidt S, Joly J, Van der Elst K, Westhovens R (2014). The performance of matrices in daily clinical practice to predict rapid radiologic progression in patients with early RA. Semin Arthritis Rheum..

[9] Aletaha D, Neogi T, Silman AJ, Funovits J, Felson DT, Bingham CO (2010). 2010 rheumatoid arthritis classification criteria: an American College of Rheumatology/European League Against Rheumatism collaborative initiative. Arthritis Rheum..

[10] Sakellariou G, Scirè CA, Zambon A, Caporali R, Montecucco C (2013). Performance of the 2010 classification criteria for rheumatoid arthritis: a systematic literature review and a meta-analysis. PLoS One..

[11] Wevers-de Boer KV, Heimans L, Huizinga TW, Allaart CF (2013). Drug therapy in undifferentiated arthritis: a systematic literature review. Ann Rheum Dis..

[12] Porter D, Dale J, Sattar N (2014). How low to aim in rheumatoid arthritis? Learning from other disciplines. Ann Rheum Dis..

[13] Arnett FC, Edworthy SM, Bloch DA, McShane DJ, Fries JF, Cooper NS (1988). The American Rheumatism Association 1987 revised criteria for the classification of rheumatoid arthritis. Arthritis Rheum..

[14] Verschueren P, De Cock D, Corluy L, Joos R, Langenaken C, Taelman V (2015). Methotrexate in combination with other DMARDs is not superior to methotrexate alone for remission induction with moderate-to-high-dose glucocorticoid bridging in early rheumatoid arthritis after 16 weeks of treatment: the CareRA trial. Annals Rheum Dis..

[15] Grigor C, Capell H, Stirling A, McMahon AD, Lock P, Vallance R (2004). Effect of a treatment strategy of tight control for rheumatoid arthritis (the TICORA study): a single-blind randomised controlled trial. Lancet..

[16] Verschueren P, Esselens G, Westhovens R (2009). Predictors of remission, normalized physical function, and changes in the working situation during follow-up of patients with early rheumatoid arthritis: an observational study. Scand J Rheumatol..

[17] Aletaha D, Funovits J, Breedveld FC, Sharp J, Segurado O, Smolen JS (2009). Rheumatoid arthritis joint progression in sustained remission is determined by disease activity levels preceding the period of radiographic assessment. Arthritis Rheum..

[18] Buttgereit F, da Silva JA, Boers M, Burmester GR, Cutolo M, Jacobs J (2002). Standardised nomenclature for glucocorticoid dosages and glucocorticoid treatment regimens: current questions and tentative answers in rheumatology. Ann Rheum Dis..

[19] Spies CM, Strehl C, van der Goes MC, Bijlsma JW, Buttgereit F (2011). Glucocorticoids. Best Pract Res Clin Rheumatol..

[20] Buttgereit F, Straub RH, Wehling M, Burmester GR (2004). Glucocorticoids in the treatment of rheumatic diseases: an update on the mechanisms of action. Arthritis Rheum..

[21] Bakker MF, Jacobs JW, Welsing PM, Verstappen SM, Tekstra J, Ton E (2012). Low-dose prednisone inclusion in a methotrexate-based, tight control strategy for early rheumatoid arthritis: a randomized trial. Ann Intern Med..

[22] Gorter SL, Bijlsma JW, Cutolo M, Gomez-Reino J, Kouloumas M, Smolen JS (2010). Current evidence for the management of rheumatoid arthritis with glucocorticoids: a systematic literature review informing the EULAR recommendations for the management of rheumatoid arthritis. Ann Rheum Dis..

[23] Duru N, van der Goes MC, Jacobs JW, Andrews T, Boers M, Buttgereit F (2013). EULAR evidence-based and consensus-based recommendations on the management of medium to high-dose glucocorticoid therapy in rheumatic diseases. Ann Rheum Dis..

[24] Meyfroidt S, van Hulst L, De Cock D, Van der Elst K, Joly J, Westhovens R (2014). Factors influencing the prescription of intensive combination treatment strategies for early rheumatoid arthritis. Scand J Rheumatol..

[25] Meyfroidt S, Van der Elst K, De Cock D, Joly J, Westhovens R, Hulscher M (2015). Patient experiences with intensive combination-treatment strategies with glucocorticoids for early rheumatoid arthritis. Patient Educ Couns..

[26] Ajeganova S, Huizinga TWJ (2015). Seronegative and seropositive RA: alike but different?. Nat Rev Rheumatol..

[27] Barra L, Pope JE, Orav JE, Boire G, Haraoui B, Hitchon C (2014). Prognosis of seronegative patients in a large prospective cohort of patients with early inflammatory arthritis. J Rheumatol..

