# Supplementary material for: Patients lacking classical poor prognostic markers might also benefit from a step-down glucocorticoid bridging scheme in early rheumatoid arthritis: week 16 results from the randomized multicenter CareRA trial
Source: Arthritis Res Ther. 2015 Apr 9;17(1):97. doi: 10.1186/s13075-015-0611-8 (PMC4422551; doi:10.1186/s13075-015-0611-8)
Supplement: Additional file 1: — List of exclusion criteria in the CareRA trial. [file 13075_2015_611_MOESM1_ESM.docx]

Supplement 1

- Previous treatment with:

o MTX or leflunomide

o Cyclophosfamide, azathioprine or cyclosporine

o SSZ for more than 3 weeks

o Hydroxychloroquine for more than 6 weeks

o Oral corticosteroids at a dosage of more than 10 mg prednisone within 4 weeks before baseline

o Oral corticosteroids at a dosage equal to or less than 10 mg prednisone within 2 weeks before baseline

o Oral corticosteroids for more than 4 weeks

o Intra-articular corticosteroids within 4 weeks before baseline

o An investigational drug for the treatment/prevention of RA.

- Contra indications for corticosteroids
- Contra indications for MTX, SSZ or Leflunomide

o Known chronic hepatic disease (alcoholic, fibrosis, …)

o Known pulmonary interstitial disease or fibrosis

o Known chronic renal failure

o History of malignant neoplasm within 5 years

o Hematologic problems at the discretion of the investigator.

- Psoriatic arthritis
- Underlying cardiac, pulmonary, metabolic, renal or gastrointestinal conditions, chronic or latent infectious diseases or immune deficiency which in the opinion of the investigator places the patient at an unacceptable risk for participation in the study.
- Pregnancy, breastfeeding or no use of a reliable method of contraception
- Alcohol or drug abuse
