# Supplementary material for: Patients lacking classical poor prognostic markers might also benefit from a step-down glucocorticoid bridging scheme in early rheumatoid arthritis: week 16 results from the randomized multicenter CareRA trial
Source: Arthritis Res Ther. 2015 Apr 9;17(1):97. doi: 10.1186/s13075-015-0611-8 (PMC4422551; doi:10.1186/s13075-015-0611-8)
Supplement: Additional file 2: — List of comorbidities in the study population and per treatment category. [file 13075_2015_611_MOESM2_ESM.docx]

Supplement 2

We have complete information about the comorbidities of patients. At baseline, the frequencies of comorbidities in the low risk population are shown in the following table:

|  | Total | TSU | Cobra Slim |
| --- | --- | --- | --- |
| Number of patients | 90 | 47 | 43 |
| Comorbidities Total | 63.30% | 66.00% | 60.50% |
| CVD | 30% | 25.50% | 34.90% |
| Cancer | 3.30% | 2.10% | 4.70% |
| Pulmonary | 6.70% | 4.30% | 9.30% |
| Diabetes | 1.10% | 2.10% | 0% |
| Gastro intestinal/ liver | 7.80% | 6.40% | 9.30% |
| Osteoporosis | 4.40% | 6.40% | 2.30% |
| Renal | 3% | 2.10% | 4.70% |
| Neurologic | 2.20% | 0% | 4.70% |
| Other | 56.70% | 59.60% | 53.50% |

TSU = Methotrexate Tight Step-Up

‘Other’ = any other comorbidities present. A full description list is available.
